# Volvulus in very-low-birth-weight preterm infants enrolled in the German Neonatal Network: prevalence, mortality, and outcome

**DOI:** 10.1186/s12887-026-06638-4

**Published:** 2026-03-12

**Authors:** Bastian Siller, Mats Ingmar Fortmann, Henry Kipke, Martina Kohl-Sobiana, Kianusch Tafazzoli-Lari, Wolfgang Göpel, Guido Stichtenoth

**Affiliations:** 1 Department of Pediatrics, University Hospital Schleswig-Holstein, University of Lübeck, Ratzeburger Allee 160, Lübeck, 23538 Germany; 2 Department of Pediatric Surgery, University of Lübeck, Ratzeburger Allee 160, Lübeck, 23538 Germany

**Keywords:** Volvulus, Very-low-birth-weight infants, Period of occurrence, Prevalence, Outcome

## Abstract

**Background:**

Volvulus is an emergency condition, but data on its prevalence and outcome in preterm infants are scarce.

**Methods:**

We analysed data from the German Neonatal Network and conducted structured literature review on the primary hospital stay of very-low-birth-weight infants who underwent surgery for volvulus. Infants who underwent surgery for focal intestinal perforation and/or necrotising enterocolitis served as a comparison group.

**Results:**

Nine relevant publications involving 97 preterm infants with volvulus were identified, revealing a wide range of postnatal onset and a mortality rate of 15.2%. The prevalence of volvulus was 123/23,652 (0.5%) according to the German Neonatal Network, which was significantly lower than the prevalence of necrotising enterocolitis and/or focal intestinal perforation. The volvulus group had a significantly higher proportion of female premature infants than the groups with necrotising enterocolitis or focal intestinal perforation. Most operations for volvulus were performed after the 20th day of life. Preterm infants who underwent surgery for volvulus had significantly less intraventricular haemorrhage and faster feeding than did those with necrotising enterocolitis and/or focal intestinal perforation. Notably, however, perioperative mortality was highest in the volvulus group. Furthermore, mortality until discharge was significantly greater in the necrotising enterocolitis group (24%) than in the volvulus group (15%).

**Conclusion:**

Volvulus occurs in five out of 1,000 very-low-birth-weight infants, particularly in those requiring immediate surgery after 20 days of age.

**Category:**

A prospective multicentre clinical cohort study.

## Introduction

In very small premature infants, most surgical abdominal interventions are performed due to focal intestinal perforation (FIP) or necrotising enterocolitis (NEC). Small bowel volvulus, on the other hand, is a rare but very serious differential diagnosis that requires immediate surgery. There are few data on volvulus at this age. An etiology due to malrotation is considered rare [1]. Although cases of gastric and sigmoideal volvulus have been reported, volvulus without any signs of malrotation is found more frequently, with an estimated incidence of 1.3 per 1,000 very-low-birth-weight infants. (VLBWI) [2]. Several risk factors for the development of volvulus without an underlying malformation have been identified in the context of preterm birth. These include a gestational age of ≤ 28 weeks [3–6], intestinal immaturity with prolonged transit time [5, 7, 8], gaseous distention due to CPAP support [5, 7, 8], intrauterine growth restriction, and female sex [5, 9].

Early diagnosis is crucial for preventing extensive intestinal damage resulting from large-scale irreversible ischemia [5, 10]. Clinical signs of volvulus include common signs of acute abdomen, such as tenderness and pain, as well as recurrent bilious vomiting, lactic acidosis and rapid deterioration of the infant’s condition [2, 4, 10]. In VLBWI, a volvulus may be misdiagnosed as a more prevalent condition, such as sepsis, FIP, or NEC. This may lead to delayed diagnosis and therapy [4].

The prognosis of volvulus depends on early diagnosis and treatment. Delayed diagnosis, particularly in cases of midgut volvulus, can have catastrophic consequences, including haemorrhage, lifelong dependence on parenteral nutrition, and associated complications [9, 11].

To better understand the circumstances surrounding the important differential diagnosis of volvulus in VLBWIs, we conducted a structured literature review. We also analysed the incidence, risk profile, age at surgery and treatment outcomes at the time of discharge after initial hospitalisation in the large database of the German Neonatal Network (GNN), which has been maintained since 2009 and contains more than 20,000 data records. We then compared these outcomes to those of infants with FIP or NEC.

## Methods

### Literature search

**Fig. 1:**
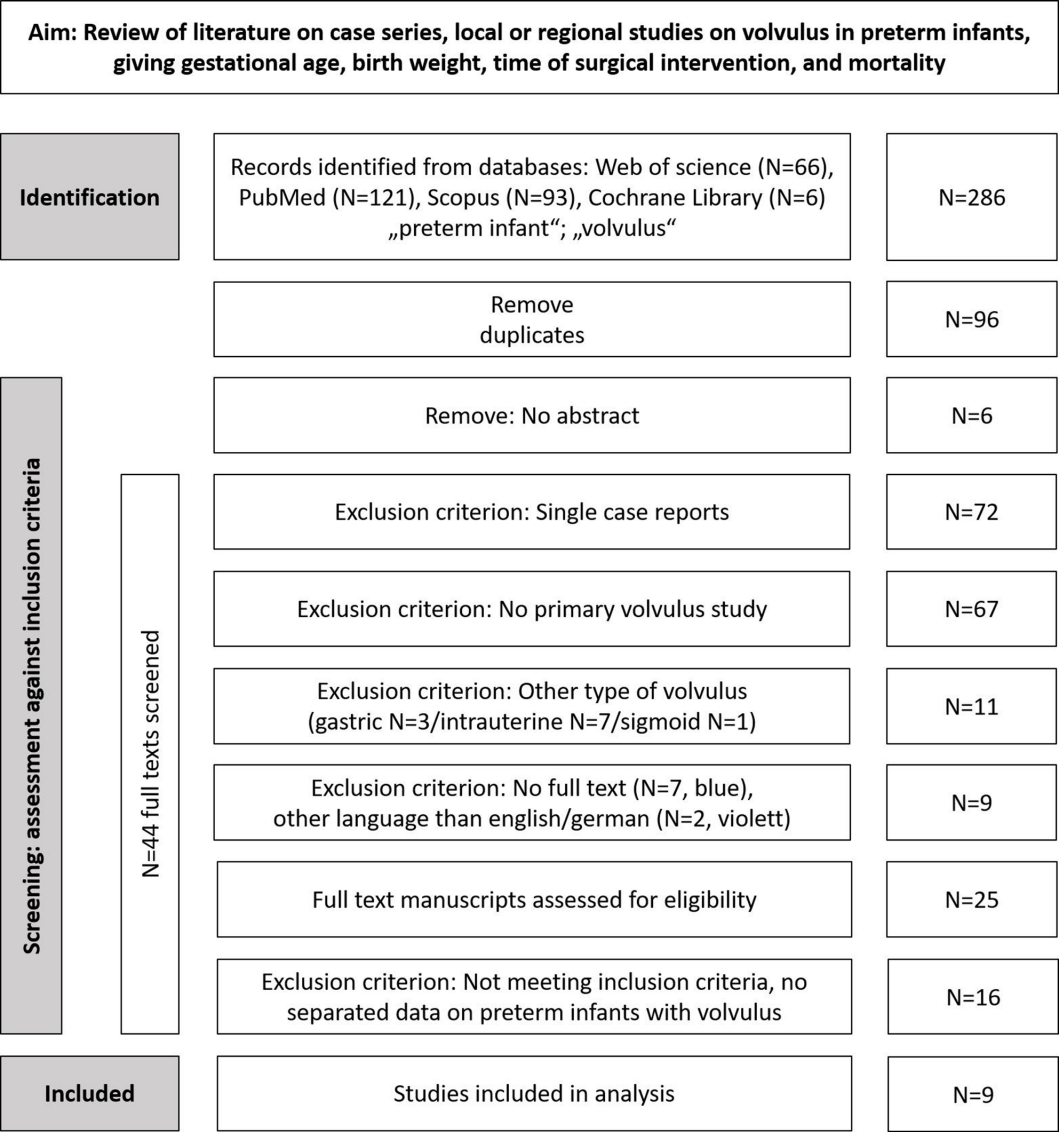
PRISMA diagram of systematic review of volvulus in preterm infants

In order to conduct a systematic review of the literature on local or regional case series concerning primary volvulus in preterm infants, a retrospective search of four databases was performed in December 2025 using the terms “volvulus” and “preterm infant”. Duplicate hits were identified and the remaining results were assessed by a reviewer using the JBI Critical Appraisal Checklist for case series [12]. The results were then described according to the PRISMA 2020 Checklist. The inclusion and exclusion criteria are outlined in Fig. 1. Records were excluded, if they were not primary volvulus studies. Systematic literature reviews, studies on imaging techniques, surgical techniques, long term outcomes or case series on volvulus following gastroschisis, congenital diaphragmatic hernias or atresia were excluded, respectively. Manuscripts describing more than one case of primary volvulus in preterm infants with a gestational age of less than 37 weeks, containing information on birth weight, age at the time of surgery and mortality, were considered.

### Patients and study design

The GNN is a national population-based observational multicentre cohort study. Currently, 71 out of 162 level 3 neonatal intensive care units in Germany are participating. Between January 2009 and December 2016, VLBWIs with birth weights of less than 1500 g and a gestational age of less than 37 + 0 weeks were enrolled. Between January 2017 and December 2019, the inclusion criteria were a birth weight of less than 1000 g or a gestational age of 28 + 6 weeks or less. Between January 2020 and December 2021, infants with a gestational age of 26 + 6 weeks or less were included. Beginning in January 2022, VLBWIs with a gestational age of 28 + 6 weeks or less were enrolled. For the present study, a subset of the GNN was selected according to the following criteria: enrolment between January 2009 and December 2023; discharge or death by 31 December 2023; and the presence of surgically treated NEC, FIP or volvulus.

Infants were enrolled after written informed consent was obtained from their parents. Predefined data on general neonatal characteristics, antenatal and postnatal treatment and outcomes were recorded by the participating centers. After discharge from primary hospitalisation, case report forms were sent to the study centre at the University of Lübeck. Data quality was evaluated by a physician or a study nurse trained in neonatology via annual onsite monitoring. The data were subsequently coded and curated for analysis. All parts of the study were approved by the ethics committees of the University of Lübeck, vote numbers 08–022 and 2023 − 812, and the participating centres.

### Definitions

The need for surgery for NEC and/or FIP was recorded by ticking a box on the case report form. As surgery for volvulus is less common, it was recorded by ticking the box ‘other surgery’ plus free text. We defined all infants who underwent surgery for volvulus as cases and infants who underwent surgery for NEC or FIP as the comparison cohort. In the event of multiple operations, only the most serious one was counted. For this purpose, surgery due to a volvulus was considered more serious than surgery for NEC, while a FIP operation was considered less serious. On the basis of national data, infants were classified ‘small for gestational age’ if their sex-specific birth weight was below the 10th percentile [13]. Bronchopulmonary dysplasia was classified if the infant received CPAP and/or oxygen for 28 days and met the criteria according to the Walsh physiological definition at gestational age 35+ 0 to 36 + 6 weeks or died of respiratory failure before that [14]. Intraventricular haemorrhages, defined by the criteria of Papile, were included at all levels of severity [15].

### Endpoints

Using the primary hospitalisation data, we compared mortality, days in hospital, weight gain and the incidence of adverse events such as intraventricular haemorrhage, blood culture-positive sepsis, bronchopulmonary dysplasia and retinopathy between the case and control groups. The daily weight gain [g/d] was calculated as (weight at discharge [g] – birth weight [g]) ÷ days in the hospital. 

### Statistics

The present study compared infants who underwent surgery for volvulus cases with a control group of infants who underwent surgery for NEC or FIP. The data were compared via the Mann‒Whitney U test for continuous variables and Fisher’s exact test for other variables. The endpoints included mortality until discharge, the duration of hospital stay until discharge, and weight gain during the hospital stay. The type I error level was set to 0.05, and the p values given were two-sided. Data analyses were performed using SPSS 29.0 (Munich, Germany).

## Results

The systematic literature review yielded 286 records (see Fig. 1). After removing duplicates and those with missing abstracts, 184 titles and abstracts were screened for inclusion criteria. Full texts of 19 cases were evaluated, and 159 records were excluded (see Fig. 1). Finally, twenty-five manuscripts were assessed in full for eligibility, and nine were selected for data extraction and summary (see Table 1). These included a total of 97 cases of volvulus recorded at different centres during the period 1981–2020. The age at the onset of volvulus ranged from a median of four to 44 days, with a minimum of zero to a maximum of 149 days. Due to missing data, a general data synthesis could not be calculated. Single data of identified cases and their outcomes were reported by Mishra [16], Kargl [17], Zweifel [18] and, partially, by Drewett [5] (*N* = 30). These patients were born with 26.7 (24–33) weeks GA, with a birth weight of 930 (475–2170) g, and the surgical intervention was at the age of 28 (2–137) days (all data: median (range)). Six publications provided data on mortality (*N* = 12 of 79 reported cases), resulting in a rate of 15.2%.

**Table 1:** Literature search

Author/Year	Cases (*N*)	GA (weeks)	[unit]	Birth weight [g]	[unit]	Age @ volvulus (days)	[unit]	Died (*N*)	Period; comment
Boulton 1989 [19]	15	34.5 ± 3.2	mean (n.s.)	2500 ± 740	mean	5 (1–35)	n.s. (R)	n.s.	1981-1985
Drewett 2009 [5]	4	32 (28–38)	M (R)	n.s.	-	4 (1–4)	M (R)	0	1996–2007; Early VWM
	6	27 (25–33)	M (R)	668 (475–2170)	M (R)	45 (22–57)	M (R)	0	Late VWM
Kargl 2015 [17]	15	26 (24–33)	M (R)	1020 (590–2040)	M (R)	20 (2–99)	M (R)	2	2003–2012; VWM
Maas 2014 [6]	5	24.4 (23.6–25.4)	M (R)	480 (370–530)	M (R)	44 (37–52)	M (R)	3	2007–2011; VWM
Horsch 2016 [20]	12	31 (24–36)	M (R)	1230 (480–2700)	M (R)	4 (1–75)	M (R)	1	2006–2013; later presenta-tion (d28-75), exclusively in 5 preterms
Mishra 2021 [16]	6	26.4 (24.6–27.4)	M (R)	770 (490–910)	M (R)	32 (25–137)	M (R)	1	2010–2020; symptomatic cases of intes-tinal malrotation in preterms < 28 weeks GA
Yarkin 2019 [2]	26	25.9 (23–32)	M (R)	728 (480–1480)	M (R)	27 (0–149)	M (R)	2	2014–2015; VWM, *n* = 6 incomplete questionaires
Montalva 2021 [21]	5	29 (n.s.)	M	1130 (n.s.)	M	24 (6–58)	M (R)	3	2017 Scientific letter
Zweifel 2013 [18]	3	27.6 (27.3–31.4)	M (R)	850 (800–1010)	M (R)	27 (26–32)	M (R)		2005–2008; Local patient chart review in cases without underlying pathology

Summary of the publications selected from a structured literature review on four databases search that yielded 286 hits. We selected those presenting volvulus case series with demographic and outcome data at the end of primary hospitalisation period

*Abbreviations*: *GA* Gestational age, *VWM* Volvulus without malrotation, *M(R)* Median(range), *n.s*. not stated

**Fig. 2:**
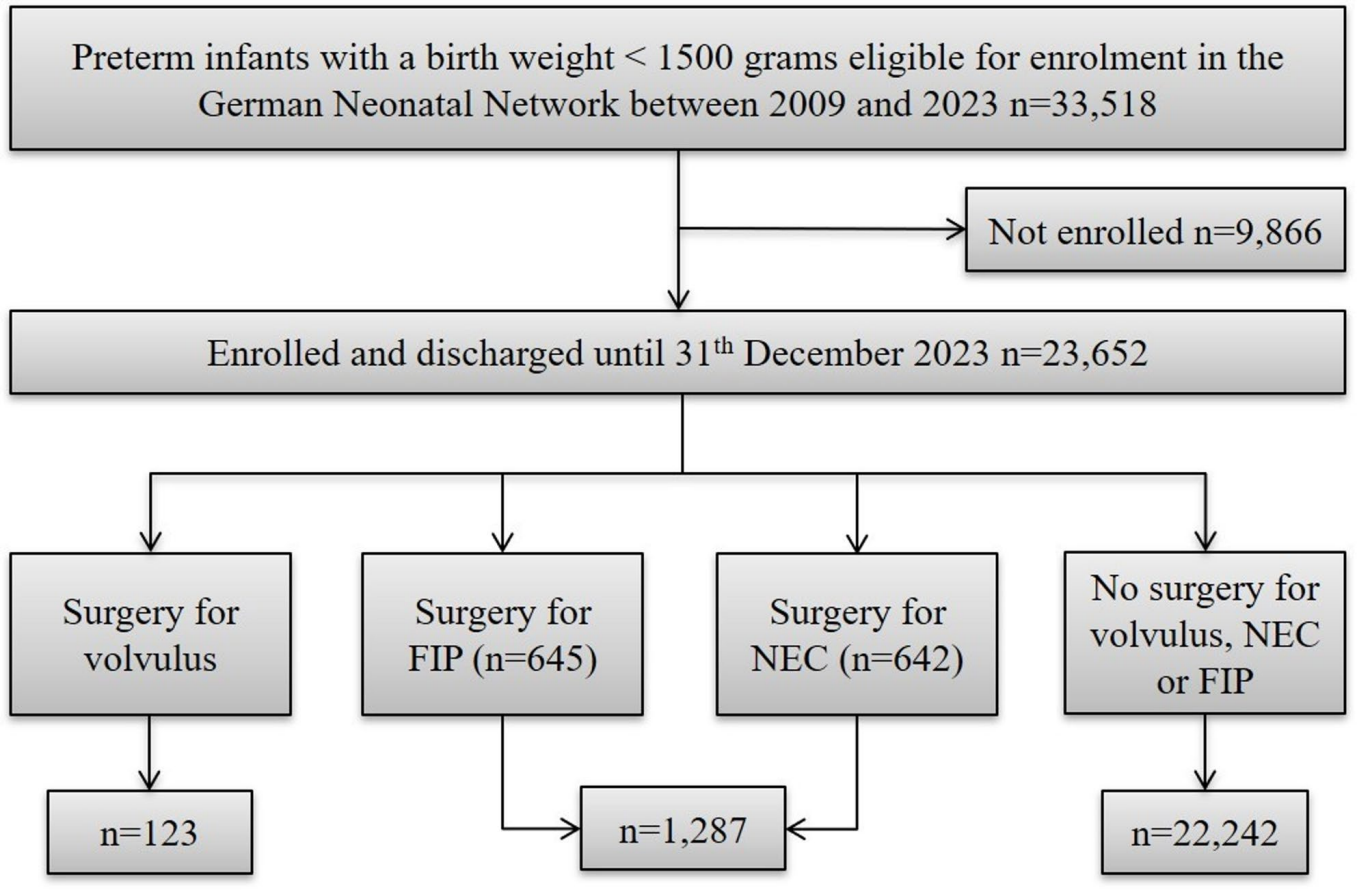
Flow chart of patient selection. Abbreviations: NEC: necrotising enterocolitis; FIP: focal intestinal perforation

Between January 2009 and December 2023, 33,518 preterm infants were eligible for enrollment in the GNN (Fig. 2). Among the 23,652 enrolled infants, the frequency of surgery for volvulus was 123 (0.5%), which was much lower than the frequency of surgery for NEC (642, 2.7%) or FIP (645, 2.7%). The infants with volvulus were slightly more mature than those in the comparison cohort were (Table 2). Compared with the NEC/FIP cohort, the volvulus cohort presented a significantly greater percentage of females. When we compared volvulus cases with the comparison cohort, we found no differences in birth weight, multiple births, the use of antenatal steroids, or the prevalence of small for gestational age.

**Table 2:** Clinical characteristics

Surgery for	Volvulus (*n* = 123)	FIP (*n* = 645)	NEC (*n* = 642)	NEC or FIP (*n* = 1287)
Gestational age (weeks)	25.6 (24.9–26.9)	24.7 (24.0–26.1) ^†^	25.4 (24.3–26.9) ^ns^	25.1 (24.1–26.4) ^†^
Birth weight (g)	715 (610–870)	670 (540–820) **	705 (580–884) ^ns^	685 (560–850) ^ns^
Female sex	65, 53%	256, 40% **	270, 42% *	526, 41% *
Multiple birth	36, 29%	247, 38% ^ns^	210, 33% ^ns^	457, 36% ^ns^
Antenatal steroids	111, 90%	586, 91% ^ns^	570, 89% ^ns^	1156, 90% ^ns^
Small for gestational age	21, 17%	147, 23% ^ns^	134, 21% ^ns^	281, 22% ^ns^
Day of surgery§	29.5 (23–51)	7 (5–12) ^†^	17 (9–28) ^†^	10 (6–20) ^†^

Summary of the clinical and demographic characteristics of patients that underwent surgery for volvulus, necrotising enterocolitis (NEC) and/or focal intestinal perforation (FIP) from the German Neonatal Network database. Categorical variables are given as n, percentage, continuous variables as median (inter quartile range). Small for gestational age was defined as less than the 10th percentile

§: Data concerning the day of surgery were available in 62 infants with surgery for volvulus and 985 infants with surgery for NEC or FIP

*P*-values were derived from Fisher’s exact test and Mann-Whitney-U test, *:*p* < 0.05, **:*p* < 0.01 and ^†^: *p* < 0.001 vs. volvulus; ^ns^: not significant

**Fig. 3:**
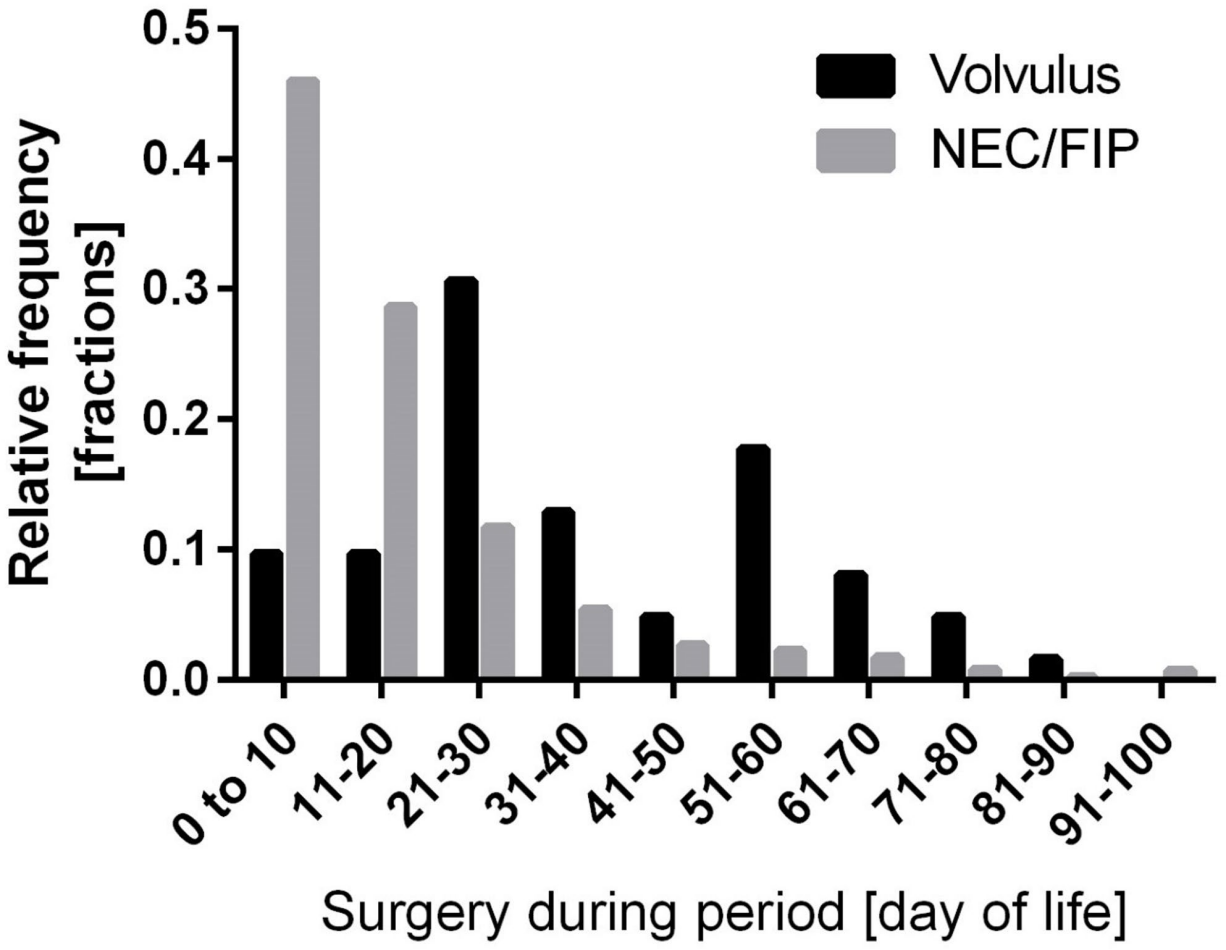
The relative frequency of infants with very low birth weights requiring surgery due to volvulus (*n* = 62) or focal intestinal perforation (FIP)/necrotising enterocolitis (NEC) (*n* = 985) is given as a fraction of the total number of cases and is shown graphically in 10-day sections up to 100 days after birth

The most significant discrepancy was observed in relation to the day of surgery. The majority of cases of volvulus occurred after 20 days of age (*N* = 49 of 62 in total; 79%), whereas the majority of cases of NEC or FIP surgery (*N* = 762 of 985 in total; 77%) were necessary within the first three weeks of life (*p* < 0.0001; Fisher’s exact test). Owing to the much higher rate of surgery for NEC or FIP, this condition was more common at any time during the hospital stay. However, the relative risk of volvulus in children who needed surgery increased over time (Fig. 3), from less than 5% within the first 20 days of life to 13–30% after that time.

Although a shorter median interval until completion of postnatal nutrition was recorded for volvulus cases than for FIP or NEC cases, interestingly, no differences were recorded in this context with respect to the cumulative total number of days with IV access (Table 3). Probiotics were used significantly less frequently in the NEC group (68%; *p* = 0.020; Fisher’s exact test) than in the volvulus group (80%). No differences were found in the prescription of carbapenems between the groups. Notably, the rate of blood culture-positive sepsis cases was significantly greater (10%) in the NEC group than in the volvulus group. However, there were no differences in the outcomes of periventricular leukomalacia, BPD or ROP. In contrast, intraventricular haemorrhages were significantly more prevalent in the NEC (40%; *p* < 0.05) and FIP (44%; *p* < 0.001; Fisher’s exact test) groups than in the volvulus group (27%). There were no significant differences in length of hospitalisation or average weight gain until discharge, although the pathological associations were different. The NEC group had the highest mortality rate (24%), which was significantly greater than that of the volvulus group (15%). However, no differences were found between the FIP group or the combined FIP/NEC group and the volvulus group. Coincidentally, perioperative mortality was significantly greater in the volvulus group (24%) than in the FIP group (9%, *p* = 0.002), the NEC group (19%, ns) and the combined FIP/NEC group (11%, *p* = 0.037; Fisher’s exact test).

**Table 3:** Short-term outcome

Surgery for	Volvulus	FIP	NEC	NEC or FIP
Data until discharge	*N* = 123	*N* = 645	*N* = 642	*N* = 1283
Intraventricular haemorrhage	33, 27%	286, 44% ^†^	253, 40% *	539, 42% ^†^
Periventricular leukomalacia	10, 8%	52, 8% ^ns^	74, 12% ^ns^	123, 10% ^ns^
Sepsis	39, 32%	203, 32% ^ns^	267, 42% *	470, 37% ^ns^
Bronchopulmonary dysplasia	59, 48%	317, 49% ^ns^	324, 51% ^ns^	641, 50% ^ns^
Retinopathy of prematurity	14, 12%	100, 16% ^ns^	119, 19% ^ns^	219, 18% ^ns^
Days with i.v. access	58 (33–103)	44 (28–73) ^ns^	62 (33–104) ^ns^	51 (30–87) ^ns^
Days until complete food tolerance	15 (11–26)	28 (20–41) *	23 (14–42) **	26 (17–41) ^†^
Use of probiotics	83; 80%	430, 75% ^ns^	396, 68% *	826, 72% ^ns^
Use of carbapenem	83, 78%	469, 82% ^ns^	487, 83% ^ns^	956, 83% ^ns^
Days in hospital	116 (79–140)	114 (88–140) ^ns^	116 (72–150) ^ns^	115 (84–144) ^ns^
Weight gain (g/d)	18.0 (14.2–21.7)	17.9 (14.6–21.2) ^ns^	17.4 (14.5–20.6) ^ns^	17.7 (14.6–20.9) ^ns^
Perioperative mortality§	15; 24%	46, 9% **	89, 19% ^ns^	135; 11% *
Mortality until discharge	19, 15%	80, 12% ^ns^	154, 24% *	234, 18% ^ns^

Outcomes of very-low-birth-weight infants who underwent surgery for volvulus, necrotising enterocolitis (NEC) and/or focal intestinal perforation (FIP). Categorical variables are given as n, percentage, continuous variables as median (inter quartile range). All other variables are given as median (inter quartile range)

§: Data concerning the perioperative mortality, defined within 30 days after first surgery, were available in 62 infants with surgery for volvulus and 985 infants with surgery for NEC or FIP

*P*-values were derived from Fisher’s exact test and Mann-Whitney-U test. ^ns^: not significant, *:*p* < 0.05, **:*p* < 0.01 and ^†^: *p* < 0.001 vs. volvulus

## Discussion

To our knowledge, the results presented here regarding volvulus in premature infants are based on the largest dataset to date. They confirm that in VLBWI, volvulus typically manifests at a later postnatal age than NEC or FIP requiring surgery. The median age at surgery was 29.5 days, which is consistent with previous studies [2, 16]. One in six operations for NEC, FIP, or volvulus in VLBWI after the 20th day of life is due to volvulus. This is of great significance for current neonatal practice.

Few large-scale studies have reported the incidence and outcome of volvulus in preterm infants. Mishra and Stringer retrospectively analysed a 10-year period in a single center study. They reported that 7/514 (1.4%) preterm infants with a gestational age of less than 28 weeks underwent surgery for volvulus. The prevalence was much higher than the 0.2% prevalence of volvulus surgery in 7,382 preterm infants at ≥ 28 weeks gestation [16]. Yarkin et al. conducted an epidemiological study including more than 20,000 VLBWIs. They reported a prevalence of 0.13%, which was higher than previous estimates based on case series [2, 22]. In our cohort, the prevalence of 0.5% for individuals with volvulus treated with surgery was within the reported range. According to our findings, girls are more likely to develop volvulus, which has been previously reported [2, 6].

In cases of extreme prematurity, the relatively early complication of FIP or NEC is associated with a 13–17% higher risk of IVH than volvulus, whereas volvulus is a relatively late event in prematurity with a high perioperative mortality risk. This means that one in four premature infants dies perioperatively, whereas only one in nine dies from FIP or NEC. However, over the period until discharge, the mortality rate for NEC appears to have reached a similarly high level as the perioperative rate for volvulus. We did not find any differences with regard to growth or weight gain compared with the comparison cohort requiring surgery for NEC or FIP. Compared to volvulus cases, infants with FIP and/or NEC achieved complete food tolerance significantly more quickly (Table 3). Further studies should investigate whether volvuli should only occur after food tolerance has been achieved.

Our study has several limitations. Detailed data on malrotation, localisation and extent of volvulus are not recorded in the GNN dataset. In addition, data on clinical symptoms preceding volvulus, such as bilious vomiting, lactic acidosis and severe abdominal pain, were not collected. Data on the day of surgery was provided for fewer than half of the volvulus cases, may have impacted the statistical analysis of the perioperative mortality. Furthermore, we cannot provide outcome data such as the need for parenteral nutrition at discharge or surgical outcome data such as transplantation, enteral autonomy, the presence of an iliocaecal valve or postoperative bowel length.

We report here that in preterm infants with a birth weight less than 1500 g, surgery for volvulus is needed for 5 out of every 1000 babies. The most striking finding of this analysis for the clinical management of VLBWI after day 20 of life is that one in six cases of abdominal surgery for NEC, FIP or volvulus suffer from the latter.

## Data Availability

The datasets used and/or analysed during the current study are available from the corresponding author upon reasonable request.

## Abbreviations

FIP: Focal intestinal perforation
GNN: German Neonatal Network
IVH: Intraventricular haemorrhage
NEC: Necrotising enterocolitis
VLBWI: Very-low-birth-weight infant
